# ﻿*Maesaflabellifera* (Primulaceae), a new species from southeast Yunnan, China

**DOI:** 10.3897/phytokeys.248.135449

**Published:** 2024-10-30

**Authors:** Dan Wei, Yuan Xu, Gang Hao, Timothy M. A. Utteridge

**Affiliations:** 1 College of Life Sciences, South China Agricultural University, Guangzhou 510642, China South China Agricultural University Guangzhou China; 2 State Key Laboratory of Plant Diversity and Specialty Crops, South China Botanical Garden, Chinese Academy of Sciences, Guangzhou 510650, China South China Botanical Garden, Chinese Academy of Sciences Guangzhou China; 3 South China National Botanical Garden, Guangzhou 510650, China South China National Botanical Garden Guangzhou China; 4 Botanical Research, Singapore Botanic Gardens, 1 Cluny Road, Singapore 259569, Singapore Singapore Botanic Gardens Singapore Singapore

**Keywords:** Ericales, Maesoideae, morphology, taxonomy, Yunnan

## Abstract

*Maesaflabellifera* (Primulaceae-Maesoideae) from southeast Yunnan, China, is described and illustrated here. This new species belongs to the informal long corolla-tube species group and is morphologically similar to *M.permollis* and *M.kurzii*, but can be distinguished by lacking hairs, membranaceous leaves and long panicles with 7−16 branches. According to the IUCN criteria, *M.flabellifera* is assessed as “Least Concern”.

## ﻿Introduction

The genus *Maesa* Forssk. (Primulaceae) contains approximately 185 species mainly distributed in tropical regions of the Old World from southern Africa through to the islands of the Pacific (Mez 1902; POWO 2024). It was originally placed in the monotypic subfamily Maesoideae of Myrsinaceae and later elevated to familial level as Maesaceae by Anderberg (2000). It is now included in an enlarged Primulaceae*s.l.* which includes four former families (Maesaceae, Myrsinaceae, Primulaceae and Theophrastaceae) and recognised as the only genus in the subfamily Maesoideae (APG 2016). According to the phylogenetic analysis, *Maesa* was found to form a basal branch, sister to all other Primulaceae species (Anderberg and Stähl 1995; Anderberg et al. 1998; Källersjö et al. 2000). Species of the genus *Maesa* are shrubs, trees or scramblers (Bremer et al. 2009) and can be distinguished from other genera of Primulaceae by the semi-inferior ovary, two bracteoles subtending each flower and black glandular lines scattered on the leaves, flowers and fruits (Sumanon et al. 2023).

Currently, approximately 35 species and two varieties (with 13 endemics) of *Maesa* have been recorded from China and they are mainly distributed in south-western China, especially in Yunnan Province. The first comprehensive revision of *Maesa* in China was conducted by Walker (1940). Chen (1979) recognised 29 species and one variety in China and divided these species into two sections, namely Maesasect.Maesa and M.sect.Doraena [Thunb.] Nakai. Chen and Pipoly (1996) reviewed the Chinese species in the Flora of China and recognised 29 species. Recently, a few taxonomic reports of *Maesa* have been published from China (Yu et al 2016; Ding et al 2023).

In February 2021, during a field survey by the first author and colleagues in Dawei Mountain National Nature Reserve in Pingbian County, Honghe Prefecture, Yunnan, an unknown species of *Maesa* in blossom was encountered and gathered. In March 2023, the same plants were discovered again in two other counties of Honghe Prefecture, namely Yuanyang and Lüchun. After a careful comparison with similar species from China and adjacent countries, it was confirmed that this species is a distinct new one and is described here.

## ﻿Materials and methods

Three field populations from Honghe Prefecture were observed and collected in February 2021 and March 2023; examination of herbarium specimens also revealed its occurrence in some other counties of Honghe Prefecture. Morphological observations were based on living plants in field and specimens deposited at IBSC and KUN. Measurements were taken with a ruler or stereomicroscope (EZ4W). The new species was compared with type specimens of similar species of *Maesa* available at the JSTOR Global Plants, as well as the specimens in BKF, E, HN, K and PE. Relevant taxonomic literature (Utteridge 2000, 2001, 2015, 2021; Utteridge and Saunders 2001, 2004; Sumanon et al. 2021, 2023) was extensively consulted. Morphological terminology follows Beentje (2016), Hewson (2019), Hickey (1979) and the Systematics Association Committee for Descriptive Biological Terminology (1962). The conservation status of the new species was assessed following the guidelines for using the IUCN Red List Categories and Criteria (IUCN Standards and Petitions Committee 2024).

## ﻿Taxonomic treatment

### ﻿ Maesa flabellifera

Taxon classificationPlantaeEricalesPrimulaceae

D.Wei, G.Hao & Utteridge sp. nov.

EFA7FDC3-DA4D-561C-B40C-824B45CB7B75

urn:lsid:ipni.org:names:77351092-1

Figs 1 , 2

#### Type.

China • Yunnan Province: Honghe Prefecture, Pingbian County, Dawei Mountain National Nature Reserve; 22.93, 103.69; 1871 m alt.; 26 February 2021 (fl.); *D. Wei et al. Xu210531* (Holotype: IBSC! barcode IBSC1025516).

#### Diagnosis.

*Maesaflabellifera* is morphologically similar to *M.permollis*, but clearly differs from the latter in the indumentum (lacking hairs vs. rusty hirsute hairs present), inflorescence structure (panicles 4.0−6.5 cm long with 7−16 branches vs. racemes or panicles 1−3 cm long with up to 3 branches). It is also similar to *M.kurzii*, but can be distinguished by the indumentum (lacking hairs vs. presence of rusty tomentose and strigose hairs) and lamina texture (membranaceous vs. chartaceous).

**Figure 1.**
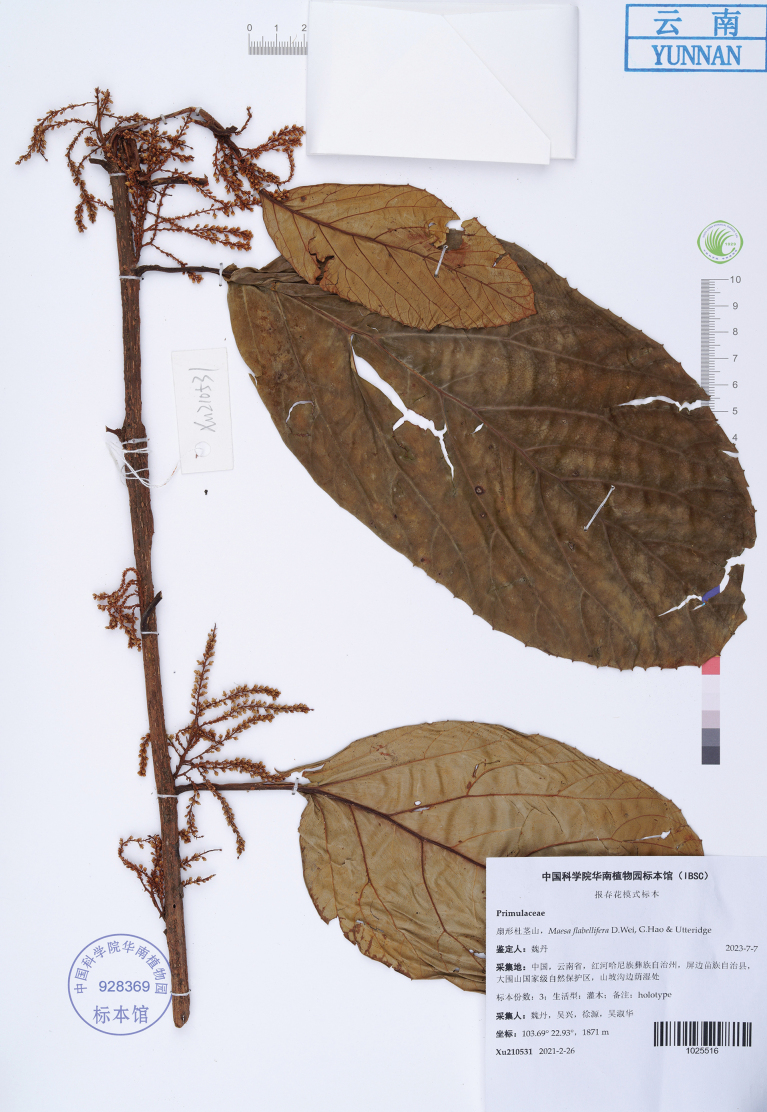
Holotype of *Maesaflabellifera* D.Wei, G.Hao & Utteridge, sp. nov. (*D. Wei et al. Xu210531*, IBSC1025516, IBSC).

#### Description.

Large shrub, up to 2.5 m tall. ***Indumentum*** all parts lacking hairs, scales present on leaves, inflorescences and fruits, scales peltate, black, ± sessile, circular with irregular margins. ***Branches*** dark green with scattered lenticels, sparsely scaly. ***Leaves*** lamina broadly elliptic to obovate, 15−35 cm long, 6−20 cm wide, membranaceous, dark green above, pale grey-green below, adaxial and abaxial surface sparsely scaly; base obtuse to cuneate; margins serrulate-serrate with 20−34 teeth on each side; apex acuminate to obtuse, sometimes emarginate; mid-rib sparsely scaly adaxially and abaxially; secondary veins 10−18 pairs, craspedodromous; densely longitudinally glandular lines; petiole 1.5−3.0 cm long, sparsely scaly. ***Staminate inflorescences*** lateral (axillary), sometimes terminal, panicles, with 7−16 branches, 4.0−6.5 cm long, axis scaly; pedicels 0.5−1.5 mm long; bracts ovate, 1.20−1.65 mm long, scaly to densely scaly, margins entire, apex acute; bracteoles ± opposite, inserted at the base of the hypanthium, triangular, 0.90−1.35 mm long, 0.4−0.6 mm wide, apex acute, margins entire, scaly. ***Staminate flowers*** pentamerous, white; calyx lobes triangular, 1.25−1.60 mm long, 0.70−1.05 mm wide, margins entire, apex acute to rounded; corolla tube 1.9−2.3 mm long, corolla lobes broadly triangular, 1.45−1.55 mm long, 1.5−1.8 mm wide, margins entire, apex rounded; stamens 5, arising 0.8−1.0 mm from the base of the corolla, filaments 1.14−1.37 mm long, anthers 0.59−0.69 mm long; hypanthium 0.75−1.20 mm long, scaly to sparsely scaly; style 1.5−2.0 mm long, stigma ± 3-lobed. ***Pistillate inflorescences and flowers*** not seen. ***Fruits*** sub-globose, ca. 3.5 mm long, ca. 3 mm in diameter, scaly to sparsely scaly; pedicels at fruiting 0.50−1.66 mm long; bracteoles remaining ± opposite at the base of the fruit; persistent calyx lobes non-overlapping.

**Figure 2.**
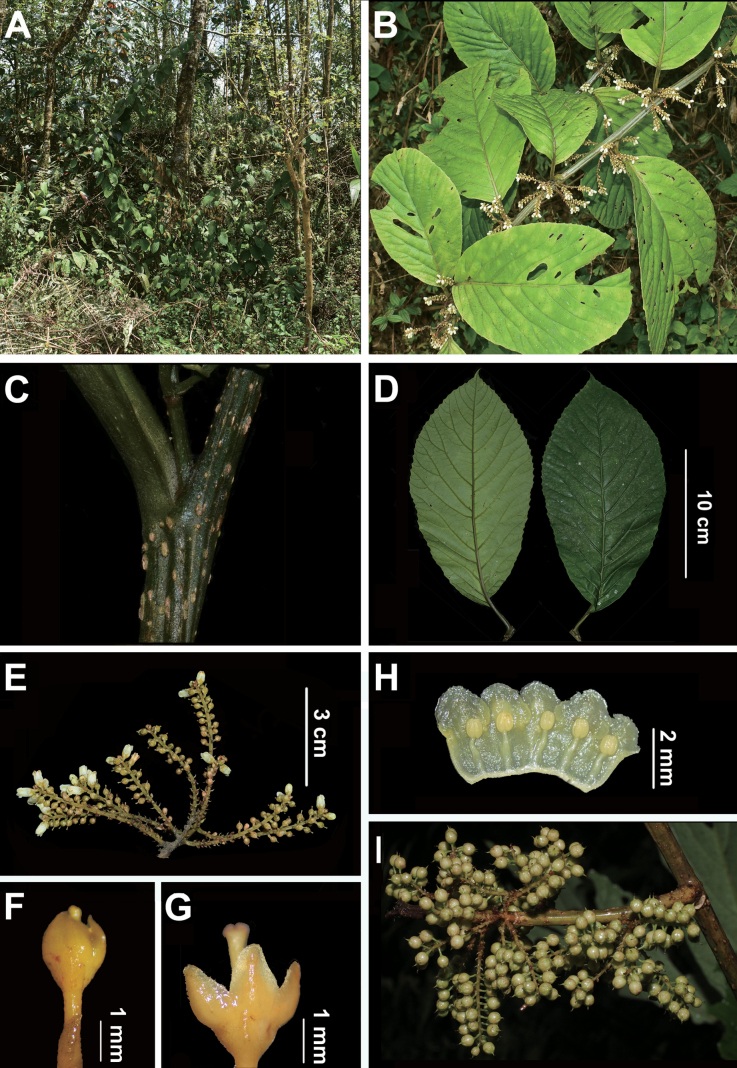
*Maesaflabellifera***A** habitat **B** habit **C** node with petiole and base of inflorescence **D** abaxial and adaxial surfaces of leaf **E** inflorescence **F** bract (borne at base of pedicel) and bracteole (borne at base of the hypanthium) **G** flower after removal of corolla **H** corolla from G, opened flat **I** Infructescences.

#### Distribution and habitat.

**Map 1.**
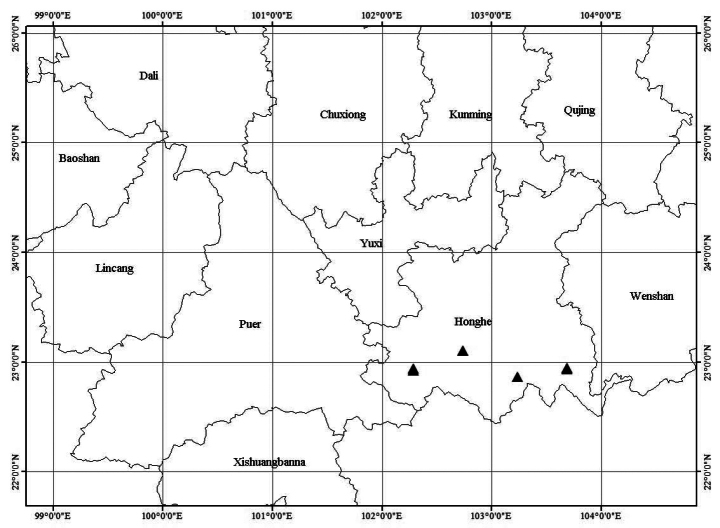
Geographical distribution of *Maesaflabellifera*.

According to the specimens examined and the recent field investigations, *Maesaflabellifera* is presently found in Honghe Prefecture, Yunnan Province (Map 1). It is common in evergreen broad-leaved mixed forests at elevations of 1500−2200 m.

#### Phenology.

Flowering from January to March, fruiting from April to December.

#### Etymology.

The specific epithet ‘*flabellifera*’ is derived from the Latin ‘flabella’ and ‘fera’ to refer to its inflorescence with 7−16 branches of almost equal length and spreading, looking like a branching fan.

#### Vernacular name.

Chinese Mandarin: shan xing du jing shan (扇形杜茎山).

#### Preliminary conservation status.

*Maesaflabellifera* is widely distributed in southeast Yunnan. In the populations in the Dawei Mountain National Nature Reserves (43993 hm^2^) and Huanglian Mountain National Nature Reserves (65058 hm^2^), the habitats are well-protected and not threatened and individuals have been found locally common in each site. Based on currently available data, *M.flabellifera* is preliminarily assessed as Least Concern (LC) according to IUCN Categories and Criteria (IUCN Standards and Petitions Committee 2024).

#### Additional specimens examined (paratypes).

China, Yunnan, Honghe Prefecture • Yuanyang County, Xinjie Town; 1891 m alt.; 22 March 2023 (fl.); *Wei et al. Xu231213* (IBSC, barcode IBSC1025520) • Lüchun County, Huanglian Mountain National Nature Reserve; 1865 m alt.; 23 March 2023 (fl.); *Wei et al. Xu231222* (IBSC, barcode IBSC1025523) • Jinping County; 2192 m alt.; 16 January 2010 (fl.); *Southeast Yunnan expedition*. *GBOWS956* (KUN, barcode KUN1279679) • Pingbian County; 1520 m alt.; 23 November 2009 (fr.); *Qian et al*. *Pbdws151* (KUN, barcode KUN1339632).

#### Notes.

Based on a phylogenetic analysis of molecular data, a new infrageneric classification of *Maesa* was proposed, dividing it into five subgenera, based on distribution and morphological characters (Sumanon et al. 2023). The species-level tree shows a strong signal of geographical distribution for the new infrageneric classification. It is speculated that *Maesaflabellifera* should be placed in the Maesasubg.Indicae Sumanon, Eiserhardt & Utteridge, by far the most species-rich subgenus in China, with species of trees or shrubs mainly from the Asian Continent, since its morphology and distribution is consistent with this clade especially the leaf morphology, such as the serrulate-serrate margins.

*Maesaflabellifera* belongs to the group of species with a longer corolla-tube. In the Flora Reipublicae Popularis Sinicae (Chen 1979), *Maesa* was divided into two sections based on the ratio of corolla-tube length to lobe length, namely Maesasect.Maesa and M.sect.Doraena [Thunb.] Nakai. There are eight species with a long corolla-tube similar to *M.flabellifera* in sect. Doraena [Thunb.] Nakai. This treatment was not adopted in the Flora of China (Chen and Pipoly 1996). Although the long corolla-tube is a good character for species-level identification as a ‘spot character’, the group is not monophyletic in the phylogenetic analysis (Sumanon et al. 2023) and is used here as a comparative tool.

*Maesaflabellifera* is unique within the long corolla-tube species group, differing from all others by the following characters: lacking hairs on all parts; leaves thick, membranaceous and broadly elliptic to obovate, 15−35 cm long and 8−20 cm wide; long paniculate inflorescences, up to 6.5 cm long, with 7−16 branches, each branch almost equal in length, looking like a branching fan arising from the leaf axils.

In the key to *Maesa* in the Flora of China (Chen and Pipoly 1996), *M.flabellifera* would key out close to *M.permollis* Kurz as they share the same leaf features and the long corolla-tube. However, *M.flabellifera* is unlikely to be confused with *M.permollis* by examination of the indumentum and inflorescence structure. Based on the herbarium and field observations, *M.permollis* is conspicuously hairy throughout with rusty hirsute hairs and the inflorescences are short, dense clustering of numerous flowers, forming compact, many-flowered inflorescence clusters. Moreover, *M.flabellifera* is found in higher elevations around 1500−2200 m, compared to *M.permollis* which is encountered at lower elevations around 500−1600 m.

Compared to the other *Maesa* species with long corolla-tubes, *M.flabellifera* is most similar to *M.kurzii*, sharing broadly elliptic to obovate leaves and long paniculate inflorescences. However, the indumentum and lamina texture make *M.flabellifera* very distinctive and easily separated from *M.kurzii*, which has chartaceous leaves, usually rusty tomentose hairs throughout and inflorescences with rusty strigose hairs. Furthermore, the distributions of these two species are distinctly different and non-overlapping. *Maesaflabellifera* is currently only known from southeast Yunnan, situated in Honghe Prefecture. *Maesakurzii* is located within Myanmar. A detailed comparison of these three species is shown in Table 1.

**Table 1.** Morphological and ecological comparison between *Maesaflabellifera* and its allies.

Features	*M.flabellifera*	*M.permollis*	*M.kurzii*
Indumentum	lacking hairs	rusty hirsute hairs	rusty tomentose and strigose hairs
Leaf texture	membranaceous	membranaceous	chartaceous
Inflorescence structure	panicles with 7−16 branches	racemes or panicles with up to 3 branches	panicles with 4−10 branches
Inflorescence length	4.0−6.5 cm	1−3 cm	3.0−4.5 cm
Elevation	1500−2200 m	500−1600 m	500−1000 m

## Supplementary Material

XML Treatment for Maesa flabellifera

## ﻿Additional information

### Author ORCIDs

Timothy M. A. Utteridge https://orcid.org/0000-0003-2823-0337

### Data availability

All of the data that support the findings of this study are available in the main text.
